# Home-based devices in dermatology: a systematic review of safety and efficacy

**DOI:** 10.1007/s00403-021-02231-0

**Published:** 2021-05-03

**Authors:** Marc Cohen, Evan Austin, Natasha Masub, Alana Kurtti, Christopher George, Jared Jagdeo

**Affiliations:** 1 Department of Dermatology, State University of New York, SUNY Downstate Medical Center, 450 Clarkson Ave, Brooklyn, NY 11203 USA; 2 Dermatology Service, VA New York Harbor Healthcare System Brooklyn Campus, Brooklyn, NY USA; 3 Rutgers Robert Wood Johnson Medical School, Piscataway, NJ USA

**Keywords:** Home devices, Hair removal, Alopecia, Light therapy, Psoriasis, Wrinkles

## Abstract

There is increasing demand for home-based devices for the treatment of dermatologic conditions and cosmesis. Commercially available devices include intense pulsed light, laser diodes, radiofrequency, light-emitting diodes, and ultraviolet B phototherapy. The objective of this report is to evaluate the current evidence regarding the efficacy and safety of home-based devices for the treatment of skin conditions. A systematic search of PubMed, Embase, and Cinahl was conducted on November 9, 2020 using PRISMA guidelines. Original research articles that investigated the efficacy and safety of home-based devices for dermatologic use were included. Bibliographies were screened for additional relevant articles. Strength of evidence was graded using the Oxford Centre for Evidence-Based Medicine guidelines. Clinical recommendations were then made based on the quality of the existing literature. After review, 37 clinical trials were included—19 were randomized controlled trials, 16 were case series, and 2 were non-randomized controlled trials. Ultimately, from our analysis, we recommend the home-based use of intense pulsed light for hair removal, laser diodes for androgenic alopecia, low power radiofrequency for rhytides and wrinkles, and light-emitting diodes for acne vulgaris. Trials investigating ultraviolet B phototherapy for psoriasis revealed mixed evidence for home treatments compared to clinic treatments. All devices had favorable safety profiles with few significant adverse events. Limitations to our review include a limited number of randomized controlled trials as well as a lack of data on the long-term efficacy and safety of each device.

**Supplementary Information:**

The online version contains supplementary material available at 10.1007/s00403-021-02231-0.

## Introduction

In recent years, dermatology has witnessed a major transition to home-based care for some cosmetic and medical problems [23]. Undesirable body hair, androgenic alopecia, acne, skin aging, and psoriasis are among the conditions with treatments that can be done at home. While these conditions were traditionally treated by dermatologists in a clinical setting, they often required frequent visits and expensive therapies [2, 3]. Given their low cost and convenience, home-based therapies are increasing in popularity [3]. For example, the hair removal industry, a $9 billion market, now consists largely of home treatments, including home devices, waxing, and depilatories [3]. The home medical equipment market accounted for $30.54 billion in 2019, and is estimated to reach $56.45 billion by 2027, with therapeutic equipment being the highest contributor [24]. Due to consumer demand, numerous Food and Drug Administration (FDA) approved products are currently on the market for home use in dermatology, many of which are light-based devices [23].

The recent advent of teledermatology may accelerate this transition to home-based care [8]. A recent review identified 229 dermatology-related mobile apps available to consumers [6]. This increase in teledermatology illustrates an emerging direction of dermatologic care that provides more patients with access to dermatologists from home [8]. We anticipate home-based devices becoming increasingly relevant as a consequence of this transition. This systematic review aims to summarize the existing literature on home-based devices in dermatology and provide evidence-based clinical recommendations on their efficacy and safety.

## Methods

**Fig. 1:**
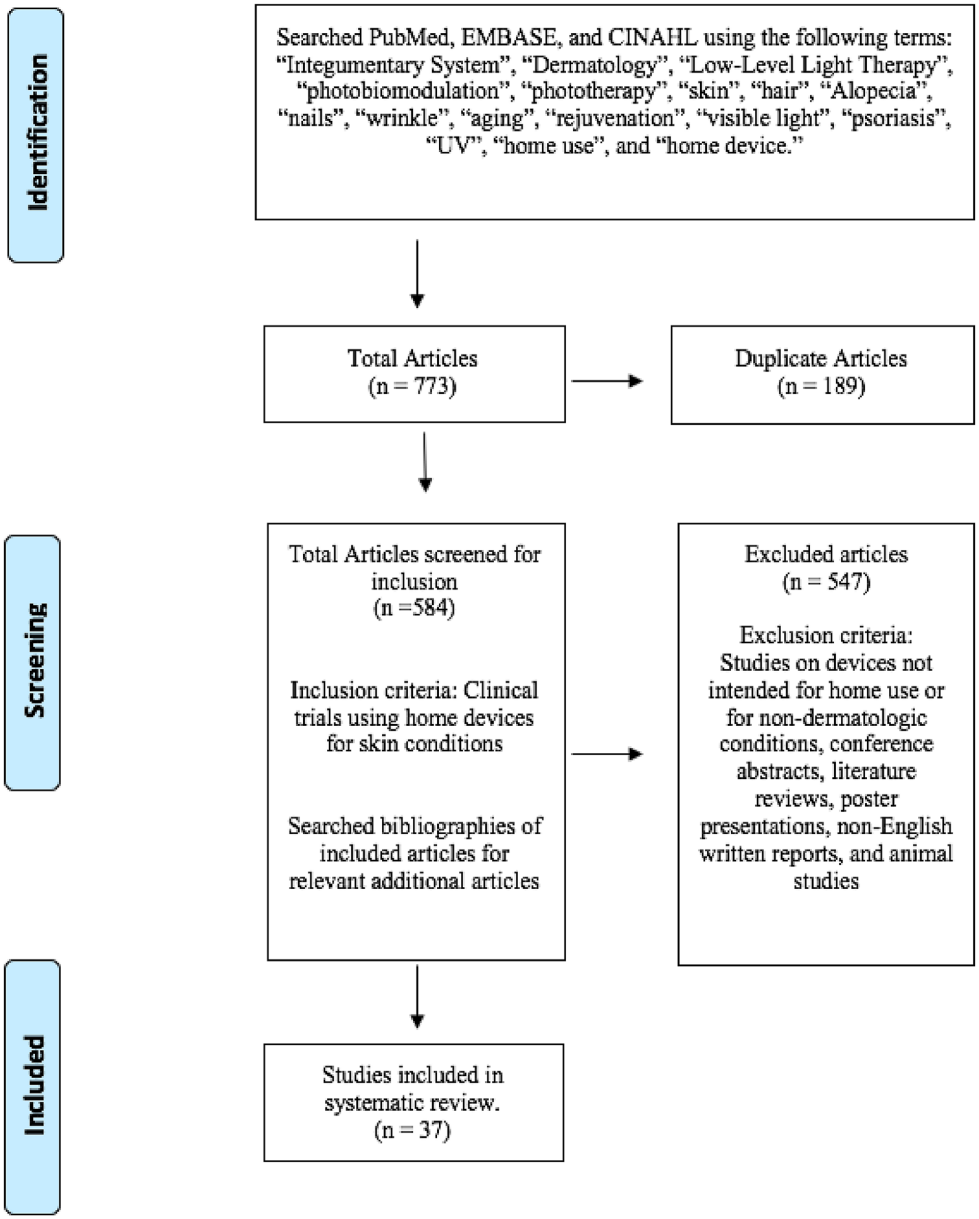
PRISMA search strategy—search conducted according to preferred reporting items for systematic reviews and meta-analysis (PRISMA) procedure

A systematic search was performed on November 9, 2020 using PubMed, Embase, and Cinahl, according to the Preferred Reporting Items for Systematic Reviews and Meta-analysis (PRISMA) guidelines. The search terms *home use* or *home device* were combined with the terms *integumentary system*, *dermatology, low-level light therapy*, *photobiomodulation*, *phototherapy*, *skin*, *hair*, *alopecia*, *nails*, *wrinkle*, *aging*, *rejuvenation*, *visible light, psoriasis*, or *ultraviolet* (Fig. 1). Search results were then screened for inclusion by two authors independently. Bibliographies of included articles were screened for additional relevant articles. Disputes between reviewers about whether a specific article fulfilled inclusion criteria had to be resolved with unanimous agreement after discussion for that article to be selected for this review. Clinical trials pertaining to the efficacy and safety of home-based devices for dermatologic indications were selected for inclusion. Studies were conducted in one of two ways: patients self-treated at home or were treated by medical professionals in a clinical setting using device parameters intended for home use. Studies that did not investigate a dermatologic indication and studies in which treatment was not intended for home use were excluded. Conference abstracts, literature reviews, poster presentations, non-English articles, laboratory investigations, and animal studies were also excluded.

## Results

We identified 670 articles regarding home use devices. After screening titles, abstracts, and full texts, 37 studies involving 1,871 patients were identified that met inclusion criteria, including 19 randomized controlled trials (RCTs), 16 case series, and 2 non-randomized controlled trials (non-RCTs). Herein, we will generally limit our discussion to RCTs identified for each home-based modality. Table I details the study design, results, and adverse effects for all studies included in this review. Level of evidence (Table 1) and grades of recommendation (Table 2) were graded using the Oxford Centre for Evidence-Based Medicine guidelines. Table 3 displays device names, manufacturers, and indications for studies referenced in the results section.

**Table 1:** Levels of evidence by study

Level of evidence	Total number of studies	Included studies
1b	16	2, 3, 12, 13, 21, 25, 26, 27, 28, 30, 31, 36, 38, 39, 40, 41
2b	19	4, 5, 7^a^, 11, 15, 17, 18, 19, 22, 29, 32, 34, 35, 37, 42, 43, 44, 45, 47
4	3	7^a^, 9, 16

Level of study is determined by Oxford Centre for Evidence-based Medicine guidelines

^a^Study 7 is included twice because it involved 2 phases of different study design

**Table 2:** Grades of recommendations

Treatment	Grade of recommendation	Number of studies by evidence level
IPL for hair removal	A	5 level 1b studies [2, 3, 30, 40, 41] 2 level 2b studies [11, 17] 1 level 4 study [9]
Laser for androgenic alopecia	A	5 level 1b studies [13, 21, 25, 28, 38] 1 level 2b study [37]
RF for wrinkles	B	1 level 1b study [31] 6 level 2b studies [4, 5, 15, 29, 34, 35]
LED for acne vulgaris	B	2 level 1b studies [27, 36] 3 level 2b studies [18, 19, 45] 1 level 4 study [16]
Phototherapy (UVB) for psoriasis	B	2 level 1b studies [12, 26] 4 level 2b studies [22, 32, 42, 47] 1 level 2b/4 study (2 phases) [7]

Grades of recommendations: A is based on consistent level 1 studies. B is based on consistent level 2 or 3 studies

*IPL* Intense pulsed light, *RF* Radiofrequency, *LED* Light emitting diode, *UVB* Ultraviolet B

**Table 3:** Device information for included studies in results section

Study number	Technology	Use	Device name	Manufacturer/location
3.30	IPL	Hair removal	*Silk’n home*	Home Skinovations, Kfar Saba, Israel
41	IPL	Hair removal	*iPulse Personal*	CyDen Ltd, Swansea, UK
2	IPL	Hair removal	*E-One device*	E-Swin, Paris, France
13	Laser	Androgenic alopecia	*HANDI-DOME*	Capillus, Miami, FL
21	Laser	Androgenic alopecia	*HairMax LaserComb*	Lexington international, Boca Raton, FL
38	Laser	Androgenic alopecia	*RAMACAP*	Division of dermatology, Ramathibodi hospital, Bangkok, Thailand
25	Laser	Androgenic alopecia	*Oaze*	Won technology, Daejeon, South Korea
28	Laser	Androgenic alopecia	*TOPHAT655*	Apira science Inc., Boca Raton, FL
31	RF	Wrinkles	*Derma Wand*	ICTV brands, Wayne, PA
34	RF	Wrinkles	*NEWA*	EndyMed medical, Caesera, Israel
27	LED	Acne vulgaris	*O’cimple Light Therapy System MP 200*	Ceragem medisys Inc., Cheonan, Korea
36	LED	Acne Vulgaris	*no!no!*	Radiancy incorporated, Orangeburg, NY
18	LED	Acne vulgaris	*Tanda Zap*	Pharos life corporation, Ontario, Canada
26	UVB	Psoriasis	*Waldmann UV-100*	Waldmann, Villingen-Schwenningen, Germany
12	UVB	Psoriasis	*Dermasun Helios*	Dermasun medical BV, Amsterdam, Netherlands
42	UVB	Psoriasis	*Clarify Home Light Therapy System*	Clarify medical, San Diego, CA

This table denotes device name, study number, technology, indication, and manufacturing information

*IPL* Intense Pulsed Light, *RF* Radiofrequency, *LED* Light Emitting Diode, *UVB* Ultraviolet B

### Intense pulsed light for hair removal

Intense pulsed light (IPL) for hair removal involves photothermolysis of hair follicles [20]. Specifically, non-coherent light of broad wavelengths (500–1200 nm) is absorbed by melanin of the hair bulb and follicle [14]. Four RCTs investigated the efficacy of IPL for hair removal [2, 3, 30, 41]. One study utilized three biweekly treatments with IPL and resulted in a 53.6% decrease in hair count at 6 months compared to baseline. Mild erythema and follicular edema were reported side effects in 25% of patients [3]. Another RCT investigating the same IPL device reported a 64% decrease in hair count at 3 months after the same treatment regimen with minimal adverse events [30]. In another IPL study involving axillary hair treatments once weekly for four weeks, there was an 87% decrease in hair count at 6 months versus control. All 10 subjects experienced a mild burning/itching sensation after administration [41]. Lastly, an RCT investigating IPL demonstrated home IPL was more efficacious and tolerable than conventional hot waxing. Mild erythema was reported [2].

Grade of Recommendation: A for the included home IPL devices for hair removal based on 5 level 1b studies, 2 level 2b studies, and 1 level 4 study (see Table 2).

### Laser diodes for androgenic alopecia

Red and near-infrared lasers can prolong the anagen growth phase of the hair follicle [38]. We identified 5 RCTs evaluating the use of home-based laser devices for androgenic alopecia [13, 21, 25, 28, 38]. One study investigating low-level laser devices demonstrated a 51% increase in hair count after laser treatments for 30 min every other day for 17 weeks compared to sham-treatments. No adverse effects were reported [13]. Another study similarly demonstrated a significant increase in terminal hair count post laser treatment relative to the control. No adverse effects were reported [21]. A study investigating laser therapy on 40 patients showed a significant increase in hair density in the laser-treated group compared to the control group. One patient reported mild hair shedding and 2 patients reported scalp pruritus [38]. Additionally, a separate RCT evaluating laser treatments once daily for 24 weeks demonstrated significantly increased mean hair density compared to the sham-treated group. Of 20 patients, 9 reported minor headache and 5 reported skin pain, pruritus, or erythema [25]. Lastly, an RCT exploring laser therapy demonstrated a 39% increase in hair count in the treatment group compared to the sham-treated group. No side effects were reported [28].

Grade of recommendation: A for the included home LED and laser devices for androgenic alopecia based on 5 level 1b studies and 1 level 2b study (see Table 2).

### Radiofrequency for rhytides and wrinkles

Radiofrequency (RF) devices stimulate fibroblast collagen production [4]. We identified 1 RCT of home-based RF for rhytides and wrinkles [31]. RF led to a maximum reduction in eyebrow to hairline distance of 1.521 cm after a single treatment. No adverse effects were reported [31]. In a related non-RCT, a home-based RF device demonstrated significant improvement in tactile elasticity and skin firmness. 45 patients received treatment 5 times weekly for 4 weeks followed by maintenance therapy twice weekly for 7 weeks. All 45 participants reported the treatment to be painless with only mild transient erythema as a side effect [34].

Grade of Recommendation: B for the included home RF devices for rhytides and wrinkles based on 1 level 1b study and 6 level 2b studies (see Table 2).

### Light emitting diode for acne vulgaris

Blue light (BL) light-emitting diodes (LEDs) activate endogenous porphyrins produced by *C. acnes* which induce free radical production resulting in bacterial cell death [18]. Red light (RL), in contrast, is believed to have an anti-inflammatory effect, attenuating cytokine release by macrophages [19]. Three RCTs investigated home-based LED devices for the treatment of acne [18, 27, 36]. One study revealed a significant decrease in inflammatory and non-inflammatory acne lesions by 77% and 54%, respectively, in 35 LED-treated patients for 2.5 min twice daily for 4 weeks. Two patients reported mild skin dryness and 1 patient experienced erythema and desquamation [27]. Another RCT demonstrated a significant decrease in time required for acne lesion resolution compared to the sham irradiated placebo. No adverse effects were reported [36]. Lastly, a study of a different LED device showed significantly decreased lesion size and degree of erythema in the treated lesions versus placebo. No adverse effects were reported [18].

Grade of recommendation: B for the included home LED devices for acne vulgaris based on 2 level 1b studies, 3 level 2b studies, and 1 level 4 study (see Table 2).

### Ultraviolet Phototherapy for Psoriasis

UVB phototherapy has demonstrated clinical efficacy for the treatment of psoriasis. The mechanism is hypothesized to involve induction of apoptosis in hyperproliferative cells, suppression of the immune response, and modification of the cytokine milieu [46]. Four RCTs evaluating home UVB phototherapy for psoriasis were included [12, 26, 32]. One study involving 3–4 treatments weekly for 3 months demonstrated a 74% decrease in median Psoriasis Activity and Severity Index score (PASI) in the home treatment group compared to a 70% decrease in clinic-treated patients. 87% of participants experienced some degree of erythema while 6% reported mild skin blistering [26]. In a separate RCT consisting of 62 patients treated for 7 min daily for 6 months, there was a significant reduction in mean PASI score at 6 months from baseline in the treatment group compared to the control group. No adverse effects were reported [12]. Another trial studying home UVB exhibited a 57% reduction in Psoriasis Severity Index (PSI) compared to no change in controls after 3 treatments weekly for 10 weeks. There were no adverse effects [42]. In contrast, in an RCT involving 40 patients, phototherapy (LISUP device) sessions given three times weekly for 8 weeks at home were compared to the same regimen provided in a clinical setting. 8 of 20 patients treated at home experienced complete clearance of psoriasis lesions compared to 18 of 20 clinic-treated patients who experienced complete clearing. Erythema was reported in both study arms [32]. The non-RCTs included in our review demonstrated efficacy for the home devices evaluated [7, 22, 42, 47].

Grade of recommendation: B for the included home phototherapy (UVB) devices for psoriasis based on 2 level 1b studies, 4 level 2b studies, and 1 level 4 study (see Table 2).

## Discussion

This analysis provides evidence for the safety and efficacy of several home-based devices for the treatment of dermatologic conditions. Home-based IPL devices demonstrated efficacy for hair removal. Reductions in hair count were consistent and exceeded 50% in all RCTs [2, 3, 30, 41]. The non-RCTs reviewed provided comparable findings. (Table I). While mild, transient erythema was the most commonly reported concern, none of the reviewed studies reported serious adverse effects [2, 3, 9, 11, 17, 30, 40, 41]. Therefore, the home-based IPL devices identified should be regarded as safe therapeutic options for patients desiring at-home hair removal. It should be noted that for darker-skinned patients, low-fluence IPL is preferred as previous research has demonstrated optimal safety at this dose [9]. Ultimately, we recommend the use of the reviewed IPL home devices for patients seeking an affordable, safe, and efficacious home hair removal therapy. The reviewed studies do not, however, provide enough evidence to recommend specific treatment parameters. Additionally, it is worth noting that the American Academy of Dermatology (AAD) has not issued specific guidelines addressing home IPL for hair removal. For completeness sake, 3 studies examining home laser devices for hair removal were included in Table I [39, 43, 44]. While laser hair removal is commercially very popular, its use for this purpose with home devices, although promising, is less well studied.

The use of home-based laser devices for the treatment of androgenic alopecia has grown over the last 15 years [38]. While vasodilator minoxidil and 5-alpha reductase inhibitor finasteride are current mainstay medical therapies, they are not effective in all patients [38]. Home-based laser devices for androgenic alopecia demonstrated increased hair growth in subjects with minimal adverse effects. Therefore, we recommend home use of this therapeutic modality with the identified devices. However, this recommendation is limited by the significant variation in treatment parameters, as adjunct LED therapy was used in some of the reviewed studies [25, 28, 38]. Therefore, a specific recommendation regarding treatment parameters and regimens cannot be made. Adverse effects were present in 3 studies but were not reported to be severe or of major concern [25, 37, 38]. For example, one patient treated with the RAMACAP device experienced minor hair shedding and two reported scalp itching (of 40 total subjects) [38]. No specific guidelines have been issued by the AAD addressing home laser devices for androgenic alopecia.

Home-based RF devices also demonstrated efficacy and a favorable safety profile for the treatment of rhytides and wrinkles. In addition to the RF studies cited in the results section, non-RCT clinical studies also exhibited statistically significant improvement in wrinkle volume and skin laxity [4, 5]. We selected a B recommendation for the RF devices included in this review. Recommended parameters include power outputs between 10 and 12 W for treatments every other day for 1–2 months. No specific guidelines have been issued by the AAD addressing home RF for wrinkle treatment.

Home-based LED devices identified in our systematic review demonstrated safety and efficacy in treating acne and received an A recommendation. In addition to the clinical studies included in our review, there is substantial basic science evidence (in vitro and in vivo studies) supporting the use of LEDs for acne treatment, and thus home devices are promising options for therapy [33]. We recommend home BL or RL LEDs with power densities of 6–40mW/cm^2^ and 8–80mW/cm^2^, respectively, and treatment regimens of 2–3 times weekly for 3–6 weeks. These suggested parameters reflect the treatment regimens identified by this review. The current AAD clinical guidelines, however, indicate that there is limited evidence to recommend the use and benefit of physical modalities, such as LED, for the routine treatment of acne [1].

The use of UV phototherapy in psoriasis has dramatically changed the landscape of psoriasis treatment options [26]. A significant drawback of UV phototherapy, however, is that it often requires numerous clinic visits which can be burdensome for patients [26]. Thus, home-based UVB phototherapy may present a convenient therapeutic option for psoriasis patients. Our systematic review identified conflicting evidence for the efficacy of home-based UVB compared to traditional clinic-based administration. In one RCT, there was statistically significant PASI score reduction with home use compared to clinic treatments while another study revealed the opposite outcome [26, 32]. Other non-randomized studies investigating home-based UVB phototherapy in psoriasis also demonstrated clinical efficacy. While the current literature leads to a B recommendation for home-based UVB phototherapy devices for psoriasis, the favorable safety profile and proof of efficacy may permit dermatologists to offer this therapeutic option for patients with medication refractory disease who prefer home treatment. However, there is insufficient evidence to make specific treatment regimen suggestions at this time. Our overall B recommendation for home-based UVB coincides with that of the AAD guidelines [10].

The major limitation of our review is the lack of double-blinded RCTs evaluating home-based devices for dermatologic treatment. Thus, many of the included studies (19 of 37) were not RCTs. While these studies do provide meaningful insights into the efficacy of these devices, they may not provide the same strength of evidence as RCTs. Additionally, most of the studies included in our review were conducted over a period of months, and thus the long-term efficacy of the devices remains uncertain. To further our understanding of the subject, double-blinded, placebo-controlled RCTs should be conducted. In addition, future studies should include long-term follow-ups to investigate the long-term effects of home-based dermatologic treatments as well as the need for maintenance treatments.

## Conclusion

Home-based devices represent the future of dermatologic treatment for a multitude of conditions given their efficacy, safety, cost-effectiveness, and convenience. We determined that home-based devices are efficacious and safe for a number of dermatologic conditions, including IPL for hair removal, laser diodes for androgenic alopecia, RF for rhytides and wrinkles, and LED-BL/RL for acne. Conflicting evidence exists regarding phototherapy for home treatment of psoriasis. All treatment modalities demonstrated favorable safety profiles. Dermatologists should consider these home-based devices to address patients’ dermatologic needs.

## Supplementary Information

Below is the link to the electronic supplementary material.

Supplementary file1 (DOCX 60 KB)

## Declarations

### Availability of data and material

All data included in this review is available in published journals.
