# Supplementary material for: Home-based devices in dermatology: a systematic review of safety and efficacy
Source: Arch Dermatol Res. 2021 May 3;314(3):239–46. doi: 10.1007/s00403-021-02231-0 (PMC8918178; doi:10.1007/s00403-021-02231-0)
Supplement: Supplementary file 1 — Supplementary file1 (DOCX 60 KB) [file 403_2021_2231_MOESM1_ESM.docx]

|  | | | | | | | | | | | |
| --- | --- | --- | --- | --- | --- | --- | --- | --- | --- | --- | --- |
| **Author** | **Device** | **Use** | **Technology** | **Patients/**  **Drop-Out** | **Study Design** | **Follow Up** | **Primary Outcome** | **Treatment Parameters** | **Treatment Regimen** | **Results** | **Adverse Events** |
| Alster, T. S.[3] | Silk'n | Hair Removal | Intense Pulsed Light (IPL) | 20/0 | Randomized Intraindividual Controlled Trial, no placebo | 1,3, and 6 months after final treatment | Hair Count | Intense pulsed light at 475 to 1,200 nm, Maximum energy of 5J (range 3–5 J/cm^2^, pulse duration < 1 ms), 2-3-cm treatment tip | Self-administered three treatments at 2-week intervals | Reduced 37.8% to 53.6% 6 months after three treatments | Mild erythema/follicular edema |
| Elm, C. M.[9] | Silk'n | Hair Removal | Intense Pulsed Light (IPL) | 10 /1 | Case Series | 4 and 12 weeks | Hair Count | Intense pulsed light at 475 to 1,200 nm, Maximum energy of 5J (Darker skin types started at lower fluence) | Once every other week for 4-6 treatments | 36% reduction after 4 weeks, 10% reduction after 12 weeks | Mild, transient erythema |
| Emerson, R. [11] | iPulse™ Personal | Hair Removal | Intense Pulsed Light (IPL) | 29/1 | Case Series | 1 and 6 months | Hair Count | Low-fluence IPL - wavelengths of 530–1200 nm using fluences of 11 J/cm^2^ and pulse durations of 15 ms, 20 ms or 25 ms depending upon skin type. A 12 x 25 mm (3 cm^2^) spot size was used | 3 sequential weekly treatments | 47% reduction at 1 month, 41% reduction at 6 months | Mild erythema |
| Gold, M. H.[17] | Silk'n Glide | Hair Removal | Intense Pulsed Light (IPL) | 17/2 | Case Series | 1 and 3 months | Hair Count | Intense pulsed light at 475 to 1,200 nm, Maximum energy of 5J | Once every other week for 6 treatments | 83.3% reduction at 1 month, 78.1% reduction at 3 months | Mild perifollicular erythema and edema |
| Mulholland, R. S.[30] | Silk’n | Hair Removal | Intense Pulsed Light (IPL) | 34/0 | Randomized Intraindividual Controlled Trial | 3 months | Hair Count | Intense Pulsed Light, wavelengths: 475 - 1200 nm, Max energy density: 5 J/cm^2^, Spot size: 20 x 30 mm, Pulse rate: 1 pulse every 3.5 seconds. | Once every other week for 3 treatments | 64% reduction | Pre-follicular erythema, Faint perifollicular edema |
| Thaysen-Petersen, D.[39] | N/A | Hair Removal | Home use laser | 36/4 | Randomized Intraindividual Controlled Trial | 1 week, 1 month, 2 months, 3 months | Hair Count, Thickness, Color | 810-nm diode laser, pulse duration of 100 ms, 1.5 cm^2^ spot size | Once every week for 8 treatments | 59% reduction, 38% thinner, and 5% lighter after 8 weeks, 29% and 7% increase in hair count and thickness at 3 months | Mild, transient erythema, discomfort, burning sensation |
| Thaysen-Petersen, D.[40] | N/A | Hair Removal | Low fluence Intense Pulsed Light | 16/0 | Blinded Physician Evaluated, Randomized Intra-individual Controlled Trial | 48 hours, 1 week, 4 weeks | Clinical skin reactions, Reflectance measurements of erythema and pigmentation, and pain | IPL: 0, 7, 8, or 10 J/cm^2^, spectral output 530–1100 nm, UVR: 3 standard erythema doses (SED’s) of solar-simulated UVR 30 min or 24 h | One treatment | No amplification of skin responses in constitutive skin of individuals with FST II–IV | Perifollicular hyperpigmentation (n=1) |
| Trelles, M. A.[41] | iPulse Personal | Hair Removal | Intense Pulsed Light (IPL) | 10/0 | Randomized Intraindividual Controlled Trial | 2 weeks, 6 months | Hair Count, Histological Evaluation | 10 J⁄cm^2^, 25 ms duration, 3 cm^2^ spots | Once every week for 4 treatments | 87% reduction | Pre-follicular erythema, burning/itching sensation |
| Wheeland, R. G.[43] | N/A | Hair Removal | Diode Laser | 132/18 | Non-randomized Controlled Trial | 1, 2, 3, 6, 9, and 12-month | Hair Count | High (22.0 J/cm^2^), medium (17.5 J/cm^2^), and low (13.0 J/cm^2^), 810 nm | TG: 3 total treatments at 3-week intervals, NTG: Single treatment | 33% reduction at 12 months | Transient Erythema |
| Wheeland, R. G.[44] | TRIA Beauty | Hair Removal | Diode Laser | 21/8 | Non-randomized Controlled Trial | 1, 2, 3, 6, 9, and 12 months | Hair Count | 7 - 20 J/cm^2^, 150 - 400 ms pulse durations, 808 nm, .81 cm^2^ spot size | 8 treatments at 1-month intervals | 44%, 49%, and 65% reduction at 12 months follow up at fluences of 7, 12, and 20 J/cm^2^, respectively | Mild/transient erythema and edema |
| Adhoute, H.[2] | E-One | Hair Removal | Intense Pulsed Light (IPL) | 63/0 | Randomized Controlled Trial | Day after treatment session | Physician assessment of efficacy and tolerance based on photographs, Physician Agreement Index | 580 nm, pulse time: 34 ms | 1 treatment every 7 weeks for 49 weeks | IPL - more efficient and tolerant | Erythema, edema, and desquamation |
| Friedman, S.[13] | HANDI-DOME LASER | Androgenic Alopecia | Diode Laser | 44/4 | Randomized Controlled Trial, Blinded Multicentered | Week 17 | Hair Count | TG: 650 nm, 272 5 mw diode lasers, 1,360 mW total delivered energy over 582 cm^2^or 2.34 mW/cm^2^, 6.92 Hz for 30 minutes, NTG: incandescent (painted) red lights | Every other day for 17 weeks for both treatment and control groups | 51% increase in hair counts as compared with sham-treated control patients | None |
| Jimenez, J.[21] | HairMax Laser-comb | Androgenic Alopecia | Diode Laser | 269/44 | Double Blinded Randomized Controlled Trial | 16 weeks and 26 weeks | Hair Count | TG: 7,9 (655 nm) for 15,11 mins, or 12 beams (6 at 635nm and 6 at 655 nm) for 8 mins. CG: Sham device - white light | Treatment three times a week for 26 weeks | Increase in terminal hair count relative to baseline in treatment group compared to control | None |
| Santino, J.[37] | Hair-Max LaserComb | Androgenic Alopecia | Diode Laser | 35/0 | Case Series | 6 months after treatment | Hair Count, Tensile Strength | N/A | 6 months of treatment | 93.5% increase in hair count, 78.9% increase in tensile strength | Early hair shedding |
| Kim, Hyojin  Choi[25] | Oaze | Androgenic Alopecia | Diode Laser | 40/11 | Double Blinded Randomized Controlled Trial | 1,12, and 24 weeks | Hair Density and Diameter | LED: 630nm (3.4 mW) and 660nm (2.5 mW), LD: 650 nm (4.1 mW), Fluence: 47.90 J/cm^2^ for 18 minutes of treatment. | Once daily for 24 weeks | Laser group showed increased hair density and mean diameter than the sham device group | Headache, skin pain, pruritus, erythema |
| Lanzafame, R. J.[28] | TOPHAT655 | Androgenic Alopecia | Diode Laser | 44/3 | Double Blinded Randomized Controlled Trial | 16 weeks | Hair Count | TG: 20, 5 mW lasers, and 31 LEDS both operating at 655nm for 25 minutes. Sham group: incandescent wheat lights that were painted red for 25 minutes | One treatment every other day for 16 weeks | 39% increase in hair count in TG relative to sham | None |
| Suchonwanit, P.[38] | RAMACAP | Androgenic Alopecia | Diode Laser | 40/4 | Double Blinded Randomized Controlled Trial | 8, 16, 24 weeks | Hair Density and Diameter | TG: 224 red laser diodes, power intensity of 3.5mW delivered over 20 mins at a wavelength of 660 nm Sham: 224 red LED pods emitting 0.5 mW of power at the wavelength of 650 nm for 20 mins | Thrice per week for 24 weeks | Hair density increase from baseline was 10.21 ± 3.25 hairs/cm^2^ in the laser group versus 3.95 ± 1.32 hairs/cm^2^ in the sham group. Hair diameter increase from baseline was 6.11 ± 2.15μm in the laser group versus 3.76 ±1.24μm in the sham group. | Mild hair shedding and scalp pruritus reported |
| Beilin, G.[4] | TriPollar STOP | Wrinkles | Radio-frequency (RF) | 23/0 | Case Series | 6 weeks, 12 weeks | Volume Analysis, Wrinkle Depth Analysis | N/A | 3 treatments per week for 6 weeks, with at least 1 day between treatments. Thereafter, subjects performed six additional maintenance treatments at 1-week intervals | Average volume reduction was 19% and 41% at the perioral and peri-orbital areas, Consistent reduction in Fitzpatrick Wrinkle Score from baseline for both perioral and peri-orbital areas at 6 and 12 weeks | Mild, transient erythema |
| Boisnic, S.[5] | POSE | Wrinkles | Radio-frequency (RF) | 24/4 | Case Series | 6 weeks, 12 weeks | Skin Laxity Rating | N/A | 2-3 times a week for two to three months | Average laxity was reduced from 1.4 at baseline to 0.8 following treatments. | None |
| Gold, M. H.[15] | Silk’n Home Skin Tightening (HST) device | Wrinkles | Radio-frequency (RF) and Light Emitting Diode (LED) Energies | 33/3 | Case Series | 1 month, 3 months | Fitzpatrick Wrinkle Score | The red and IR LEDs emit optical power of 70 and 55 mW/cm^2^, respectively; the RF generator provides 1 MHz RF with maximal output power of 10W, reaching a maximal temperature of 43°C | Every other day for 6 weeks (treatment phase) followed by two monthly maintenance treatments during the next 12 weeks (follow-up phase) | Average reduction of 1.49 Fitzpatrick scores for patient wrinkle assessment | Erythema, Edema |
| Leyden, J.[29] | PaloVia | Wrinkles | Non-Ablative Fractional Laser | Pilot Study) 36/2 Pivitol Study) 100/10 | Case Series | Pilot Study) Week 4, 8, 12, 16, 20, 28 Pivitol Study) Week 2, 4, 8, 12 | Photoassesment and Fitzpatrick Wrinkle Score | Center wavelength range: 1410 nm, Output window dimensions: 9 by 13 mm, Energy: 8-15 mJ, Pitch: 0.9-2.0 mm | Pilot Study) Daily self-treatments were performed for 4 weeks followed by 4 weeks of twice-per-week maintenance treatments. Pivitol Study) Daily self-treatments were performed for 4 weeks followed by 12 weeks of twice-per-week maintenance treatments. | Mean FWS scores decreased from 5.1 and 4.5 to 4.0 and 3.3 for Pilot and Pivotal subjects, respectively | Erythema, Edema, Skin roughness, dryness, bronzing, flaking, and itching |
| Nobile, V.[31] | DermaWand | Wrinkles | Radio-frequency (RF) | 50/0 | Double Blinded Randomized Placebo Controlled Trial | No follow up | Eyebrow Lifting Effect - Eyebrow to hairline distance measurement | 14-16 kW, 10 microsecond burst, 50x/second on 220 volt power device and 60x/second on 110 volt power device, RF energy of 100kHz over 6 minutes | One-time treatment | Decreased eyebrow to hairline distance in treated versus control group when compared to baseline | None |
| Sadick, N. S.[34] | NEWA | Wrinkles | Radio-frequency (RF) | 47/2 | Case Series | Weeks 4, 8, and 12 | Visual Assessment Score for wrinkles, brightness, elasticity, lift, firmness, tone, smoothness, and luminosity | 12W of phase-controlled RF energy through six electrodes | 5 times a week on facial skin for weeks 1-4, then 2 times a week for weeks 5–12 | Most participants had clinically significant improvement in the texture, luminosity, tactile elasticity, and firmness of their skin as well as a facial and jaw skin lift | Mild, transient erythema |
| Sadick, N. S.[35] | NEWA | Wrinkles | Radio-frequency (RF) | 69/7 | Case Series | Weeks 1, 2, 3, 4, 1 month, and 3 months | Fitzpatrick Wrinkle and Elastosis Scale | 12W of phase-controlled RF energy through six electrodes | At least 5 treatments a week, for one month | Improvement (downgrade of at least 1 score according to the Fitzpatrick scale) in 91.93%, 96.77%, and 98.39% of study subjects (according to the first, second, and third reviewer, respectively) | None |
| Gold, M. H.[16] | Silk’ n Blue | Acne Vulgaris | Light Emitting Diode (LED) | 17/4 | Case Series | 1 month and 3 months | Lesion Count | LED BL (405-460 nm) | 8 treatment sessions twice a week over a period of 4 weeks | Decrease in mean acne vulgaris lesion counts from 16.33 at baseline to 10.58 at the 1-month follow-up to 6.45 at the 3-month follow-up visit. | None |
| Gold, M. H.[18] | Tanda Zap (TZ) | Acne Vulgaris | Light Emitting Diode (LED) | 30/0 | Randomized Intraindividual Controlled Trial | Until 10 days after first treatment or blemish resolves | Lesion Size and Degree of Erythema | LED BL (414 nm) | 2 treatments/day for 2 consecutive days | 37% complete clearance of lesions treated with the active unit as opposed to only 10% clearance achieved with the placebo at the end of treatment. Also, decreased size and erythema in TG versus placebo | None |
| Kwon, H. H.[27] | O' cimple Light Therapy System MP 200 | Acne Vulgaris | Light Emitting Diode (LED) | 35/0 | Double Blind, Randomized Controlled Trial | Weeks 2, 4, 8, and 12 | Acne grade, number of lesions, and histopathology | LED BL (420 nm, 6.1mW/cm^2^, 0.91J/cm^2^) and LED RL (660 nm, 8.1mW/cm^2,^ 1.22J/cm^2^) for 2.5 minutes | 2 treatments/day for 4 weeks | Inflammatory and noninflammatory acne lesions had decreased significantly, by 77% and 54%, respectively, in the treatment group. No significant difference was observed in the control group. | Mild transient dryness, erythema, and desquamation |
| Sadick, N.[36] | no!no! | Acne Vulgaris | Light and Heat energy (LHE) based systems | 63/2 | Double Blind, Placebo Controlled, Randomized Control Trial | Day 5 | VAS and PLRS scores | Broad spectrum light (450 - 2000 nm), Fluence level - 6 J/cm^2^ | Twice a day for 4 days: once a day at home and once a day at the clinic in front of an unblinded observer | Statistically significant shorter lesion improvement and lesion resolution rates | None |
| Goldberg, D. J.[19] | Omnilux (blue and revive) | Acne Vulgaris | Light Emitting Diode (LED) | 24/2 | Case Series | Weeks 2, 4, 8, and 12 | Lesion Count | LED BL (415 nm wavelength, 40 mW/cm^2^ intensity, total dose 48 J/cm^2^ after 20 minute exposure), LED RL(633 nm wavelength, 80 mW/cm2, total dose 96 J/cm^2^ after 20 minute exposure) | 2 treatments/week for 4 weeks, for a total of 8 treatments | Mean lesion count reduction was significant at week 4 (46%) and week 12(81%) | Mild erythema |
| Wheeland, R. G.[45] | Handheld Blue Light Device | Acne Vulgaris | Light Emitting Diode (LED) | 32/1 | Case Series | Weeks 1, 2, 3, 4, 6, and 8 | Lesion count; number, severity, and redness of flares | LED BL (412nm), Area A: 29J/cm^2^/day Area B:2J/cm^2^/day | 2 treatments/day for 8 weeks | Median reductions in inflammatory lesion count at Weeks 1, 4, and 8 were 29, 43, and 60 percent, respectively, in Area A, and 23, 33, and 46 percent, respectively, in Area B. | Minimal transient dryness, hyperpigmentation |
| Cameron, H.[7] | HoPE | Psoriasis | Phototherapy (UVB) | Phase 1) 10/3 Phase 2) 23/0 | Case Series | N/A | Presence of Psoriatic Plaques | Phase 1) UVB (10.38 J/cm^2^ for 25-40 minutes) Phase 2) UVB (9.84 J/cm^2^ for 25-40 minutes), .95kW | Phase 1) 3 treatments a week for approximately 6 weeks 2) 3 treatments a week for approximately 10 weeks | Phase 1) Seven patients completed therapy and reached MRA (Minimal Residual Activity) or clearance Phase 2) 18 patients reached MRA or clearance and 3 showed moderate improvement | Erythema |
| Franken, S. M.[12] | Dermasun Helios | Psoriasis | Phototherapy (UVB) | 62/17 | Randomized Controlled Trial | 2, 4, 6 months | PASI score | Low emission UVB treatment for 7 minutes | Daily use for 6 months | Significant reduction in mean PASI score at 6 months from baseline in treatment group versus control | None |
| Jordan, W. P., Jr.[22] | N/A | Psoriasis | Phototherapy (BB-UVB) | 56/1 | Case Series | End of treatment | Presence of Psoriatic Plaques | 1-8 minutes of UVB (150-170 microW/cm^2^) | 7 days/week for 6-8 weeks | 100% of the patients had 99% clearance | None |
| Koek, M. B.[26] | Waldmann UV-100 | Psoriasis | Phototherapy (narrowband UVB (TL-01)) | 196/18 | Randomized Single Blind Multi Center Controlled Study | 23 and 46 irradiations, and then 2, 4, 6, 8, 10, 12 months | PASI score, SAPASI score | UVB (TL-01) cumulative dose of 51.5 J/cm^2^ | 3/4 treatments/ week for approximately 3 months | Patients treated at home: median SAPASI score decreased 82% (from 6.7 to 1.2) and the median PASI score decreased 74% (from 8.4 to 2.2), compared with 79% (from 7.0 to 1.4) and 70% (from 7.0 to 2.1) for patients treated in an outpatient setting. | Mild/severe erythema, burning sensation, blistering |
| Paul, B. S.[32] | LISUP | Psoriasis | Low Intensive Selective Phototherapy (UVB) | 40/0 | Randomized Controlled Trial | N/A | Presence of Psoriatic Plaques | UVB (300-320nm) for five minutes at the first treatment and increasing three minutes at each additional treatment | 3 treatments/week for approximately 8 weeks | Home: 8/20 cleared and 5/20 improved Outpatient:18/20 cleared and 1/20 improved | Erythema |
| Unrue, E. L.[42] | Clarify Home Light Therapy System | Psoriasis | Phototherapy (narrowband UVB) | 14/6 | Randomized Intraindividual Controlled Trial | Weeks 4, 6, 8, and 10 | PSI Score | UVB (300-320 nm), 4.5cm x 4.5cm target | 3 treatments/week for 10 weeks | 100 % patients experienced improvement in the treated lesions, with a mean improvement of 57% in PSI (P<0.0001 compared to baseline and P<0.0002 compared to the control lesions). | None reported |
| Yentzer, B. A.[47] | Panosol II | Psoriasis | Phototherapy (NB-UVB) | 27/5 | Case Series | Week 12 | Patient Adherence to treatment | Narrow Band UVB treatment | 3 treatments/week for 12 weeks | Adherence rates to home phototherapy were higher than adherence rates for Acitretin | None reported |

Table I. Home Devices in Dermatology

Table I Legend:

**BB – UVB** – Broadband Ultraviolet B

**BL –** Blue light

**FST –** Fitzpatrick Skin Type

**FWS -** Fitzpatrick Wrinkle Score

**IPL –** Intense Pulsed Light

**LED –** Light Emitting Diode

**LHE –** Light and Heat Energy

**MRA –** Minimal Residual Activity

**N/A –** Not applicable

**NB –** UVB – Narrowband Ultraviolet B

**NTG –** Non-treatment Group

**PASI -** Psoriasis Area and Severity Index

**PLRS -** Photographic Lesion Reference Scale

**RL –** Red light

**SAPASI -** Self Administered Psoriasis Area and Severity Index

**SED –** Standard Erythema Doses

**TG –** Treatment Group

**VAS -** Visual Analogue Score
